# Acute fibrinous and organizing pneumonia in a patient with Sjögren’s syndrome and *Legionella* pneumonia: a case report and literature review

**DOI:** 10.1186/s12890-022-01997-x

**Published:** 2022-05-24

**Authors:** Ye Lu, Wei Zheng, Wei Cao, Xianghong Yang, Li Zhao, Yu Chen

**Affiliations:** 1 Department of Pulmonary and Critical Care Medicine, Shengjing Hospital of China Medical University, Shenyang, China; 2 Department of Pathology, Shengjing Hospital of China Medical University, Shenyang, China

**Keywords:** Acute fibrinous and organizing pneumonia, Sjögren’s syndrome, *Legionella* pneumonia, Case report

## Abstract

**Background:**

Acute fibrinous and organizing pneumonia (AFOP) is a rare clinicopathological condition. Studies in the literature have reported that AFOP may be associated with respiratory infections, such as respiratory syncytial virus, influenza virus, *Pneumocystis jirovecii*, *Penicillium citrinum*, and *Chlamydia* infections. However, AFOP associated with *Legionella* infection has not been reported previously. Here, we report a case of a patient with AFOP secondary to Sjögren’s syndrome and *Legionella* infection.

**Case presentation:**

A 47-year-old man was admitted to the hospital because of fever, expectoration, and shortness of breath. Lung imaging showed irregular patchy consolidation. A diagnosis of *Legionella* pneumonia was initially considered on the basis of the patient’s history of exposure to soil before disease onset, signs of extrapulmonary involvement, and a positive *Legionella* urine antigen test result. However, the patient’s symptoms and lung imaging did not improve after treatment with levofloxacin, moxifloxacin, and tigecycline for *Legionella* infection. In addition, Sjögren’s syndrome was diagnosed on the basis of clinical manifestations and immunological indicators. Pathological changes associated with AFOP were confirmed from the results of ultrasound-guided percutaneous lung biopsy. The patient’s clinical symptoms improved rapidly after a short course of low-dose corticosteroid therapy, and lung imaging showed significant improvement.

**Conclusions:**

The possibility of secondary AFOP should be considered when *Legionella* pneumonia does not improve after standard antibiotic therapy. Lung biopsy and histopathological examination are important for the adjustment of treatment strategy. Our case also highlights the importance of screening for autoimmune diseases in patients with AFOP.

## Background

Acute fibrinous and organizing pneumonia (AFOP) is a recently recognized rare clinicopathological condition. The histopathological characteristics of AFOP include large amounts of fibrin deposition in the alveolar cavities and changes associated with organizing pneumonia (OP) [1]. One type of AFOP has an acute onset and progresses rapidly, leading to multiple organ failure and death, with a clinical course similar to that of diffuse alveolar damage (DAD). The other type of AFOP has a subacute onset and a clinical course similar to that of cryptogenic organizing pneumonia (COP) [2]. The causes of AFOP are unclear. The onset may be related to infection, autoimmune disease, adverse drug reactions, and environmental exposures [3]. Pathogens reported to be associated with AFOP include *Haemophilus influenzae*, *Acinetobacter baumannii*, *Chlamydia*, respiratory syncytial virus, influenza virus A/H1N1, human immunodeficiency virus, and *Pneumocystis jirovecii*, *Penicillium citrinum* et al. [1, 4–9]. To our knowledge, there have been no reports of AFOP associated with *Legionella* infection. To improve the clinical understanding of AFOP, we report a case of a man with Sjögren’s syndrome and *Legionella* infection in which the lung pathology was consistent with that of AFOP.

## Case presentation

**Fig. 1:**
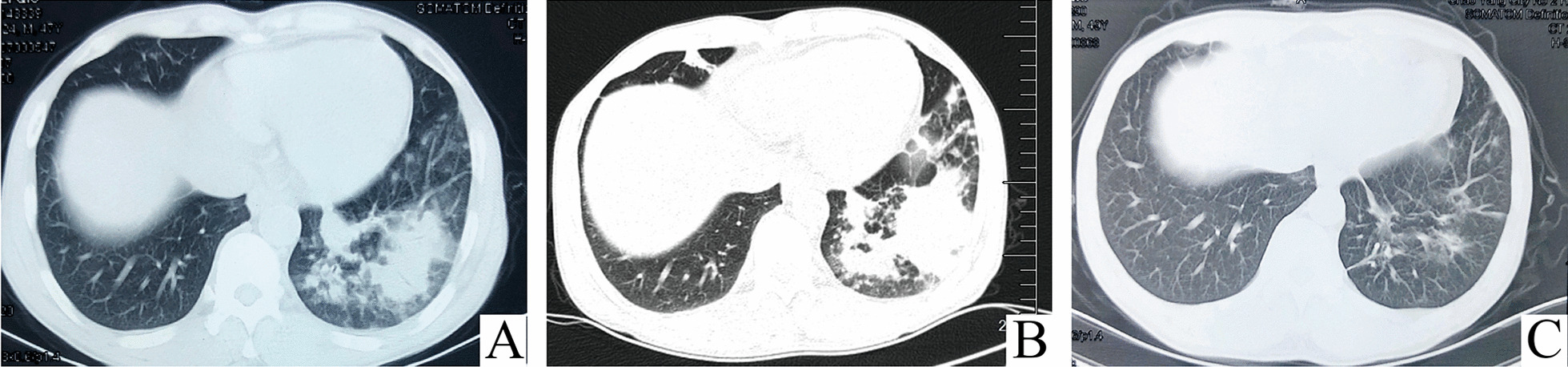
Computed tomography (CT) of the patient’s chest showing progression of the pulmonary lesions. **a** Chest CT performed at a local hospital prior to admission to our hospital showing irregular patchy opacities in the left lower lobe. **b** Chest CT performed on admission to our hospital showing extension of the patchy opacities in the left lower lobe since the previous scan, and new small patchy opacities in the right middle lobe. **c** Follow-up chest CT performed after 1 month of corticosteroid treatment showing almost complete resolution of the pulmonary lesions

A 47-year-old Chinese male farmer, with history of contact with soil during farm work 2 days before the onset of illness, was admitted to our hospital with a 2-week history of a high fever, productive cough with purulent sputum, shortness of breath on exertion, and nausea and vomiting. Chest computed tomography (CT) performed at a local hospital before admission to our hospital had shown irregular patchy opacities in the lower lobe of the left lung (Fig. 1a). He had been treated with levofloxacin for 4 days and then switched to moxifloxacin and meropenem for 10 days at the local hospital. However, as his fever, expectoration, and shortness of breath had not improved, he was transferred to our hospital for further investigation and treatment.

The patient had a 7-year history of dry mouth and dry eyes which had not previously been medically evaluated or treated, but had no history of other possible autoimmune disease such as recurrent oral ulcers, Raynaud’s phenomenon, rash, or arthritis. He was a smoker with a 30 pack-year smoking history. He denied a history of pulmonary disease. He didn’t undergo any previous chest X-ray examination and reported no symptoms and signs of previous interstitial lung disease such as gradually progressive dyspnea on exertion, dry cough, fatigue of unknown origin. He also denied a history of occupational dust exposure, keeping pets and drug abuse. He had no family history of pulmonary disease and cancer.

On admission, he had a temperature of 37.6 °C, pulse of 82 beats/min, respiratory rate of 16 breaths/min, and blood pressure of 123/71 mmHg. The skin on both his legs was dry and rough, but there was no rash or bruising. There were no rales in the lungs on auscultation, and no other obvious abnormalities were noted on physical examination.

The chest CT images on admission at our hospital showed that the patchy opacities in the left lower lobe had extended since the previous chest CT, and there were new small patches in the right middle lobe (Fig. 1b). Laboratory tests revealed an elevated white blood cell count and neutrophil percentage, elevated levels of procalcitonin and C-reactive protein, an increased erythrocyte sedimentation rate, mild hyponatremia, and elevated levels of liver enzymes. The serum human immunodeficiency virus antibody was negative. No infectious agents were detected in the patient’s blood culture and sputum microbiological examination (Table 1).

**Table 1:** Main Laboratory test

Test	Day 1 (admission)	Day 8	Discharge	Reference result/range
*Hematology*				
White blood cell count (× 10^9^ cells/L)	20.59	11.53	5.78	3.5–9.5
Neutrophil percentage (%)	87	81.7	57	42.5–71.5
Procalcitonin (ng/mL)	0.117			< 0.05
C-reactive protein (mg/L)	103	43.1	12	0–8
Erythrocyte sedimentation (mm/hr)	100			0–15
*Liver enzymes*				
Alanine transaminase (U/L)	101			0–40
Aspartate transaminase (U/L)	60			5–34
Alkaline phosphatase (U/L)	463			40–150
Gama-glutamyl transferase (U/L)	209			9–64
Serum sodium (mmol/L)	132			136–145
*Infections*				
*Legionella* urine antigen	Positive			Negative
*Streptococcus pneumoniae* Urine antigen	Negative			Negative
Blood culture	No growth			No growth
Sputum microbiological examination	Negative			Negative
Serum anti-*Mycoplasma* IgM	Negative			Negative
Serum anti-*Chlamydia* IgM	Negative			Negative
Serum anti-*Legionella* IgG	Negative			Negative
Serum HIV antibody	Negative			Negative
*Immunological test*				
Tear secretion test (mm/5 min)	Left eye: 3; right eye: 9			10–15
Antinuclear antibodies	Positive, titer 1:160			Negative, < 1:80
Anti-SSA/Ro60	5.2 (positive)			< 0.8 negative
Anti-SSA/Ro52	5.5 (positive)			< 0.8 negative
Anti-SSB/La	0.8 (weak positive)			< 0.8 negative
Anti-histone	0.9 (weak positive)			< 0.8 negative
Rheumatoid factor (IU/mL)	94.5			0–30

*HIV* human immunodeficiency virus, *IgM* Immunoglobulin M, *IgG* Immunoglobulin G

Because the patient had symptoms and signs of extrapulmonary system involvement, including gastrointestinal symptoms, hyponatremia, and signs of liver damage, we could not rule out *Legionella* pneumonia and thus we performed a *Legionella* urine antigen test, which was positive. The patient was treated with moxifloxacin 0.4 g once a day and piperacillin sodium and tazobactam sodium 4.5 g three times a day for 8 days. His white blood cell count, neutrophil percentage and C-reactive protein level had decreased since admission (Table 1). However, his symptoms of fever, expectoration, and shortness of breath persisted. Thus, the antibiotic treatment was switched to intravenous tigecycline 50 mg twice a day for 5 days after an initial dose of 100 mg. However, his symptoms still did not improve. To confirm the diagnosis, we performed an ultrasound-guided percutaneous needle lung biopsy and tested for markers of autoimmune disease.

The result of a tear secretion test was positive. Salivary gland emission computed tomography dynamic imaging showed impaired uptake in both submandibular glands and parotid glands. Immunological index testing gave positive results for antinuclear antibodies, anti-SSA/Ro60, anti-SSA/Ro52, anti-SSB/La and anti-histone antibody, and the serum rheumatoid factor level was increased (Table 1). Test for autoimmune liver disease-related antibodies, antineutrophil cytoplasmic antibodies, were all negative, and the serum complement C3 and C4 levels were normal.

**Fig. 2:**
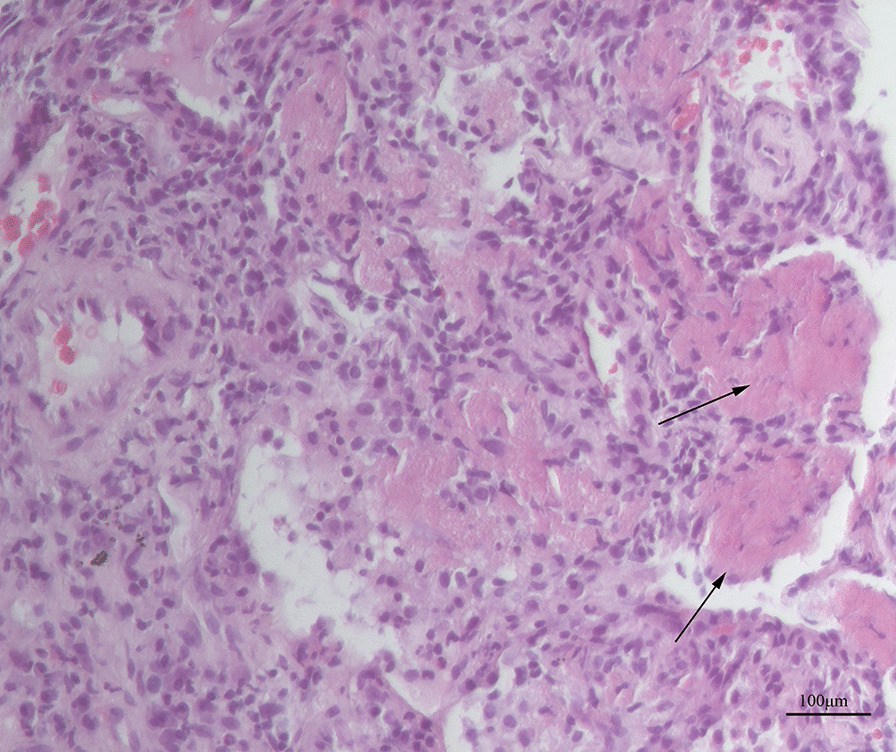
Histopathology of the lung tissue. Fibrin balls are present in the alveolar cavities (arrows), and the alveolar septa are widened, with infiltration of chronic inflammatory cells and fibrosis. (Hematoxylin and eosin stain; magnification × 200). Picture was taken by ZEISS Microscope Model AXIO Lab A1, ZEISS camera Model Axiocam ICc 5 and ZEN software at a resolution of 96dpi, and processed in adobe photoshop 21.0.2 at a resolution of 300 dpi. No downstream processing was utilized

Lung histology showed formation of fibrin balls in the alveolar cavities accompanied by changes associated with OP (Fig. 2). No pathogens were detected in the lung tissue using metagenomic next generation sequencing (mNGS). Based on the results of the lung biopsy, antibiotics were stopped and intravenous methylprednisolone 40 mg daily was initiated. The patient’s symptoms improved rapidly, and his body temperature returned to normal on the first day of corticosteroid therapy. He was discharged home 4 days later, and continued oral prednisone 10 mg daily for 20 days followed by 5 mg daily for 5 days. At the follow-up visit one month after discharge, his chest CT showed almost complete resolution of the pulmonary lesions (Fig. 1c).

## Discussion and conclusions

The concept of AFOP was first proposed by Beasley et al. [1] to describe a class of pathological tissue types that does not conform to the clinicopathological manifestations of DAD, OP, or eosinophilic pneumonia. AFOP is a disease that pathologically manifests as the formation of fibrin balls in alveolar cavities, accompanied by the development of OP. In terms of course and prognosis, there are two main types of AFOP: a type with an acute or fulminant course leading to respiratory failure with rapid progression to death and a type with a subacute course with a generally good prognosis. Our patient’s condition conformed to the latter type. It is still debated whether AFOP should be classified as an independent disease, but AFOP was classified as a rare histologic pattern in the 2013 edition of the classification of idiopathic interstitial pneumonias by the American Thoracic Society/European Respiratory Society [10]. The clinical manifestations of AFOP lack specificity and primarily manifest as shortness of breath, cough, and fever. AFOP may be related to numerous clinical conditions, such as infections, autoimmune diseases, adverse drug reactions, and environmental exposures (Table 2) [1, 3–9, 11–13]. There are no significant differences in the clinical and lung imaging manifestations of AFOP according to the cause. AFOP associated with different conditions exhibits a number of common clinical manifestations and the characteristics usually overlap with those of the primary disease.

**Table 2:** The clinical conditions associated with AFOP [1, 3–9, 11–13]

Unknown	Idiopathic
Infections	Haemophilus influenzae, Acinetobacter baumannii, Chlamydia, respiratory syncytial virus, influenza virus A/H1N1, human immunodeficiency virus, Pneumocystis jirovecii, and Penicillium citrinum
Autoimmune diseases	Ankylosing spondylitis, antisynthetase syndrome, collagen vascular disease, fibromyalgia, juvenile dermatomyositis, dermatomyositis, polymyositis, primary biliary cirrhosis, very severe aplastic anemia, undifferentiated connective tissue diseases, systemic lupus erythematosus, necrotizing myopathy, and primary Sjögren’s syndrome
Durgs	Abacavir, amiodarone, bleomycin, decitabine, everolimus, sirolimus, zacytidine
Environmental exposures	Aerosols, asbestos, coal, dusts
Others	Myelodysplastic syndrome, lung transplant, bone marrow transplant

*AFOP* acute fibrinous and organizing pneumonia

Beasley et al*.* [1] reported that the amount of chronic inflammatory cell infiltration surrounding the fibrin balls in AFOP lesions varies and noted that AFOP may be associated with lung infection. AFOP may improve following antibiotic treatment [14], suggesting that infection may trigger the onset of AFOP. Previous reports have shown that a variety of infections may be associated with AFOP, including bacterial infections (*Haemophilus influenzae*, *Acinetobacter baumannii*), viral infections (respiratory syncytial virus, influenza virus A/H1N1, human immunodeficiency virus), and fungal infections (*Pneumocystis jirovecii*, *Penicillium citrinum*) [1, 5–9]. In the reported case, *Legionella* pneumonia was diagnosed based on a history of contact with soil before the onset; symptoms of fever, expectoration and shortness of breath; extrapulmonary manifestations (gastrointestinal symptom, liver function injury and hyponatremia), and the positive *Legionella* urine antigen test result. The reason that mNGS did not reveal *Legionella* in lung tissue was probably because the infection was already under control because of the early use of antibiotics effective against *Legionella*. Based on the result of the mNGS, the patient was treated with corticosteroid alone following discharge and remained in a stable condition.

AFOP and OP have some imaging and pathological manifestations that are common to both. Previous studies have found that *Legionella* pneumonia can cause changes associated with OP [15]. Haroon et al*.* [15] suggested that alveolar epithelial cell injury caused by *Legionella* infection and subsequent tissue repair are involved in the pathology of OP and the development of its imaging manifestations. After alveolar injury, proteins and serous fluid in the capillaries enter the alveolar cavity, where they may be involved in fibrin ball formation [14]. This may explain the AFOP manifestations in the lungs of this patient.

*Legionella* infection often manifests as severe pneumonia, with a mortality of 8–12% [16]. In nosocomial cases, the mortality can be as high as 34% [16]. When patients with *Legionella* pneumonia have poor response to treatment and their condition worsens, clinicians may consider whether the antibiotics are adequate, but rarely consider the possibility that the worsening may be due to the development of OP or AFOP as a complication. It has been reported that AFOP may have a case fatality rate of more than 50% [1]. Corticosteroids are the first-line treatment for AFOP, and some patients require corticosteroid pulse therapy. The use of corticosteroids during the acute stage of treatment for *Legionella* pneumonia is not recommended and corticosteroids should not be used routinely in patients with *Legionella* pneumonia. Therefore, in patients with *Legionella* pneumonia who do not respond to adequate antibiotic treatment, if the clinical features and imaging suggest AFOP, timely lung biopsy is very important to make the diagnosis of secondary AFOP, such that the treatment can be changed accordingly, leading to an improved prognosis.

In this patient, the possibility of AFOP related to Sjögren’s syndrome must also be considered, as the course of corticosteroid therapy was decided on the basis of the possibility that the AFOP was secondary to Sjögren’s syndrome. Previous studies have found that the pathological types of interstitial lung lesions caused by Sjögren’s syndrome include non-specific interstitial pneumonia and usual interstitial pneumonia, with some cases manifesting as OP and lymphocytic interstitial pneumonia [17–19]. Chest CT imaging typically shows predominantly ground-glass opacities, linear opacities, interlobular septal thickening, cysts, reticulation, and nodules [18–22]. Consolidation opacities similar to those observed in community-acquired pneumonia are rare. So far, including our case, a total of 3 cases of Sjögren’s syndrome with AFOP have been reported (Table 3) [13, 23]. The lung imaging abnormalities in those two previous reported patients consisted primarily of nodules or nodular consolidation with or without ground-glass opacities. One of the patients was treated with immunosuppressive agents in addition to corticosteroids. Our patient’s lung imaging was reported to show patchy consolidation, which differed from the usual pulmonary involvement associated with Sjögren’s syndrome. Sjögren’s syndrome is a relatively indolent connective tissue disease, and not all patients with Sjögren’s syndrome require corticosteroid and/or immunosuppressive therapy. According to the European League Against Rheumatism criteria for assessment of Sjögren’s syndrome [24], if our patient’s lung changes were secondary to Sjögren’s syndrome, this would be consistent with moderate Sjögren’s syndrome, and a medium-to-high-dose corticosteroid therapy would be required, with tapering and maintenance at a low dose before discontinuation. Treatment with immunosuppressive agents may also be required in patients with AFOP secondary to autoimmune disease. Wang et al. [13] reviewed 13 cases of AFOP associated with autoimmune disease and found that most cases required immunosuppressive therapy in addition to corticosteroid therapy. However, our patient required only a short course of low-dose corticosteroid therapy, and his symptoms and imaging rapidly improved. In addition, although the patient didn’t undergo previous chest X-ray, he denied a history of pulmonary disease and lacked risk factors, symptoms and signs of interstitial lung disease. Furthermore, the patient had experienced symptoms of Sjögren’s syndrome for many years before the onset of AFOP, without respiratory symptoms, suggesting that the AFOP is most likely to have been secondary to *Legionella* infection rather than Sjögren’s syndrome. It is possible that Sjögren’s syndrome played a synergistic role with *Legionella* infection in the pathogenesis of AFOP in our patient.

**Table 3:** Summary of reported cases of Sjögren’s syndrome with AFOP patients including present case

References	Age/sex	Other trigger conditons	Clinical presentation	Chest radiography	Method of diagnosis	Treatment	Outcome
[13]	60/Female	No special	Acute (fever, dyspnea, non-productive cough)	Nodules admixed with GGO	CT guide needle lung biopsy	Corticosteroid	Improved
[23]	75/Female	No special	Acute (shortness of breath, wheezing, productive cough)	Multilobar consolidation	Transbronchial lung biopsy	Corticosteroid, mycophenolate mofetil, hydrochloroquine	Improved
Present case	49/Male	*Legionella* pneumonia	Acute (fever, shortness of breath, productive cough)	Irregular patchy consolidation	Ultrasound-guided needle lung biopsy	Corticosteroid	Improved

*AFOP* acute fibrinous and organizing pneumonia, *GGO* ground-glass opacity, *CT* computerized tomography

In summary, we reported a case of AFOP secondary to Sjögren’s syndrome with *Legionella* infection. Although AFOP is a rare clinicopathological disorder, the possibility of AFOP secondary to *Legionella* pneumonia should be considered in patients with *Legionella* pneumonia who do not respond to antibiotic therapy and in patients with Sjögren’s syndrome with acute onset of respiratory symptoms, with or without infectious conditions. Pathological examination should be conducted as early as possible if patients with *Legionella* pneumonia do not respond to appropriate antibiotic treatment, and corticosteroids should be administered to improve prognosis. Our report also highlights the importance of screening for autoimmune diseases in patients with AFOP.

## Data Availability

All data and material analyzed during this study are included in this published article.

## Abbreviations

AFOP: Acute fibrinous and organizing pneumonia
COP: Cryptogenic organizing pneumonia
CT: Computed tomography
mNGS: Metagenomic next generation sequencing
OP: Organizing pneumonia
